# Identification and functional analysis of C-type lectin from mosquito *Aedes albopictus* in response to dengue virus infection

**DOI:** 10.1186/s13071-024-06453-9

**Published:** 2024-09-04

**Authors:** Sheng Gao, Haodong Xu, Hongbo Li, Xiao Feng, Jitao Zhou, Renxian Guo, Zihan Liang, Jinying Ding, Xin Li, Yijia Huang, Wenquan Liu, Shaohui Liang

**Affiliations:** Department of Medical Parasitology, School of Basic Medical Sciences, Wenzhou Medical University, Wenzhou, 325035 Zhejiang China

**Keywords:** *Aedes albopictus*, C-type lectin, Expression patterns, Functional study, Dengue virus

## Abstract

**Background:**

C-type lectins (CTLs) are a large family of proteins with sugar-binding activity. CTLs contain an evolutionarily conserved C-type lectin domain (CTLD) that binds microbial carbohydrates in a calcium-dependent manner, thereby playing a key role in both microbial pathogenesis and innate immune responses. *Aedes albopictus* is an important vector for transmitting dengue virus (DENV) worldwide. Currently, the molecular characteristics and functions of CTLs in* Ae. albopictus* are largely unknown.

**Methods:**

Transcripts encoding CTL proteins in the *Ae. albopictus* genome assembly were analyzed via sequence blast. Phylogenetic analysis and molecular characterization were performed to identify the functional domains of the CTLs. Quantitative analysis was performed to determine the gene expression features of CTLs during mosquito development and in different tissues of female adults after blood feeding. In addition, the functional role of CTLs in response to DENV infection was investigated in *Ae. albopictus* mosquito cells.

**Results:**

We identified 39 transcripts encoding CTL proteins in the *Ae. albopictus* transcriptome. *Aedes albopictus* CTLs are classified into three groups based on the number of CTLDs and the domain architecture. These included 29 CTL-Ss (single-CTLDs), 1 immulectins (dual-CTLD) and 9 CTL-Xs (CTLDs with other domains). Phylogenetic analysis and structural modeling indicated that CTLs in *Ae. albopictus* are highly conserved with the homologous CTLs in *Aedes aegypti*. The expression profile assay revealed differential expression patterns of CTLs in both developmental stages and in adult female tissues. Knockdown and overexpression of three CTLs (CTL-S12, S17 and S19) confirmed that they can promote dengue virus infection in *Ae. albopictus* cells.

**Conclusions:**

The CTL genes in *Ae. albopictus* mosquito and other mosquito species are evolutionarily conserved and exhibit different developmental and tissue expression features. The functional assay indicated that three CTLs in *Ae. albopictus* mosquitoes are involved in promoting dengue virus infection. Our study revealed that CTLs play important roles in both the physiological processes and viral infection in mosquito vectors.

**Graphical Abstract:**

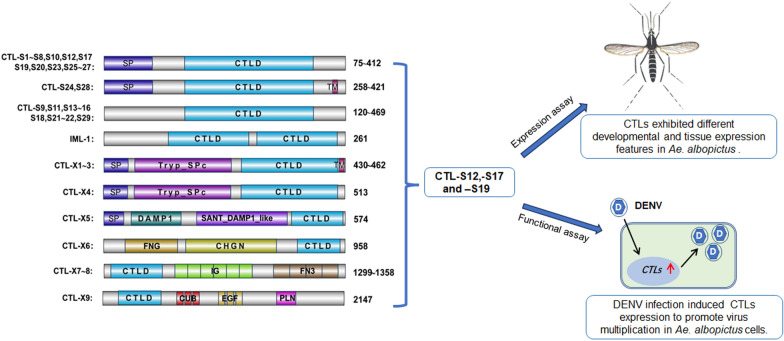

**Supplementary Information:**

The online version contains supplementary material available at 10.1186/s13071-024-06453-9.

## Background

The epidemic of mosquito-borne viruses is a global health burden [1]. *Aedes albopictus* is an important vector for transmitting several important mosquito-borne viruses, including dengue, chikungunya and Zika viruses [2, 3]. Dengue virus (DENV) infection in humans causes a broad spectrum of clinical symptoms, including dengue fever (DF), dengue hemorrhagic fever (DHF) and dengue shock syndrome (DSS) [4]. The World Health Organization (WHO) estimates that there are more than 300 million DENV infections per year worldwide, but no vaccines or therapeutics are available [5, 6]. Currently, mosquito control is the only effective strategy for preventing dengue transmission [7, 8].

The replication and proliferation of DENV must be completed in mosquito vectors before it is successfully transmitted to vertebrate hosts [9]. As the virus-infected blood meal is ingested into the midgut, DENV first infects midgut epithelial cells and is then disseminated into the salivary glands and other tissues via the hemolymph [10, 11]. DENV infection in mosquitoes is usually associated with low fitness costs, thereby allowing infected mosquitoes to transmit the virus efficiently [9]. Vector competence during virus infection is mainly determined by unique antiviral mechanisms, including RNAi and innate immune signaling (Toll, IMD and JAK-STAT) [12]. Innate immune responses are initiated by the recognition of “non-self” components on the surface of pathogens, known as pathogen-associated molecular patterns (PAMPs) [13]. Insects have developed a variety of pattern recognition receptors (PRRs) to recognize and bind to PAMPs of foreign microorganisms [13–15].

C-type lectins (CTLs) constitute a large family of PRRs that have been identified in both invertebrates and vertebrates [16, 17]. CTLs usually bind to carbohydrates in a calcium-dependent manner via carbohydrate-recognition motifs in C-type lectin domains (CTLDs) [17]. The sugar-binding specificity of CTLs is determined by several conserved residues in CTLDs [18]. CTLs containing Glu-Pro-Ser/Asn (EPS/N) or Gln-Pro-Asp (QPD) motifs in the CTLD are responsible for mannose- or galactose-binding activity, respectively [19]. Many genes encoding CTL proteins have been identified in *Drosophila melanogaster* [20], *Manduca sexta* [21], *Bombyx mori* [22] and *Aedes aegypti* [23]. CTLs in arthropods have numerous biological functions including cell adhesion, pathogen sensing and the initiation of immune responses [18, 24].

Previous studies have demonstrated that mosquito CTLs play an important role in regulating parasites and virus infection [23, 25–27]. Knockout of CTL4 in *Anopheles gambiae* can enhance melanization-based refractoriness to the *Plasmodium* ookinetes, indicating that CTL4 acts as an essential mosquito-encoded factor for malaria parasites transmission [25]. *Aedes aegypti* CTLs (mosGCTLs) were significantly upregulated by DENV, West Nile virus (WNV) and Japanese encephalitis virus (JEV) infection and facilitated viral infection in vivo and vitro [23, 26, 28]. Blood feeding female mosquitoes with antisera against mosGCTLs dramatically reduced the arbovirus infection, indicating that CTLs can be used as the targets for developing a transmission-blocking strategy against mosquito-borne diseases [26–28].

In this study, we described the molecular characterization of the CTL protein families in *Ae. albopictus.* We identified 39 putative CTL proteins in the *Ae. albopictus* genome assembly and characterized the expression patterns of three CTLs during mosquito development and in the tissues of female adults following the consumption of blood meals. Furthermore, we investigated the functional role of CTLs in response to DENV infection in *Ae. albopictus* cells. These results revealed that CTLs not only are involved in physiological processes but also play an important role in regulating DENV infection in *Ae. albopictus*.

## Methods

### Identification and characterization of CTL proteins in *Ae. albopictus*

To determine the number of transcripts encoding CTL proteins in the *Ae. albopictus* genome, we searched every possible CTL-coding sequence according to the CTL homolog genes from *Ae. aegypti* [23]. The CTL domain (CTLD) and transmembrane regions were predicted by using the SMART tool. The processing sites of the signal peptides (SPs) were predicted by applying the SignalP 5.0 online tool. The domain architectures were plotted with Photoshop CS5.0 version. The gene ID and sequence information of CTL-coding genes were retrieved from VectorBase (Additional file 2: Table S1).

### Sequence alignments and phylogenetic tree construction

The CTL sequences derived from *Ae. aegypti* and *Culex quinquefasciatus* were aligned with putative CTLs from *Ae. albopictus* using Clustal X. The phylogenetic tree was constructed via the neighbor-joining method using the MEGA X software package as described before [7]. The amino acid alignments of C-type lectins were performed by using the ESPript 3.0 online tool. The conserved amino acids in CTLD were identified by comparison to the sequences of the CTL superfamily in other insects [21, 22].

### Structure modeling of mosquito CTLs

Amino acid sequences of CTLD proteins from *Ae. albopictus* and *Ae. aegypti* were submitted to the Iterative Threading ASSEmbly Refinement (I-TASSER) server (http://zhanglab.ccmb.med.umich.edu/I-TASSER/) for predicting and refining 3D structures. Structural templates were first identified from PDB by the multithreading program LOMETS. Full-length models were then constructed via iterative simulations. The generated PDB files were rendered and visualized using the PyMOL Molecular Graphics System.

### Mosquito rearing, blood feeding and tissue dissection

Mosquito *Aedes albopictus* (Foshan strain) rearing and blood feeding of female adults were performed as described previously [7]. In brief, adult mosquitoes were maintained at 28 °C and 80% humidity with a 12:12 h light:dark cycle. Ground yeast and 10% sucrose solution were provided for larvae and adult mosquitoes, respectively. Tissue samples from eggs, larvae (L1 to L4 stages), pupae and adults (female and male) were collected to examine the temporal expression of CTL genes. Female adults were starved overnight before blood feeding. Artificial blood was purchased from JUSHI BioTech. (HENAN YC16086). Mosquitoes were allowed to feed the blood meals containing 1 mM ATP by using an artificial membrane feeder with a 37 °C water jacket for 2 h. Only fully engorged mosquitoes were used for the subsequent tissue dissection according to the previous methods [29]. Then, the tissue homogenate was centrifuged at 12,000 × g for 5 min at 4 °C, and the supernatant was transferred to a new tube for RNA extraction.

### Cell culture and DENV infection

*Aedes albopictus* C6/36 cells (ATCC, CRL-1660) were cultured in RPMI-1640 medium (Gibco) supplemented with 10% FBS and antibiotics (100 units/ml penicillin and 100 mg/ml streptomycin). C6/36 cells were maintained at 28 °C in a 5% CO_2_ atmosphere. The DENV2 New Guinea C strain (ATCC VR-1584™) was propagated in the C6/36 cells, and the vial titers were determined by plaque assay as described previously [29].

For DENV infections in the study, 1 × 10^6^ C6/36 cells were seeded into one well of a 12-well plate for culturing overnight. DENV infection were carried out as described before with minor modification [26]. C6/36 cells were infected with DENV2 at a multiplicity of infection (MOI) of 1 for 2 h. The cells were washed with 1 × PBS buffer to remove uninternalized virus. The infected cells were cultured in the fresh growth medium from 24 to 48 h and were collected for RNA and protein extraction.

### RNA extraction and RT-qPCR

RNA extraction was performed by using TRIzol reagent (Invitrogen) according to the manufacturer’s instructions. The isolated RNA was treated with RNase-free DNase and used as the template for reverse transcription-quantitative PCR(RT-qPCR). RT-qPCR was performed by using ChamQ SYBR qPCR Master Mix (Vazyme Biotech, Nanjing, China) according to the manufacturer's instructions in an ABI QuantStudio 6 Pro Real-Time PCR System (Applied Biosystems, CA). The ribosomal S7 (*rps*7) gene was used as the reference gene. Relative gene expression at different developmental stages was assessed via normalization to the levels of the *rps*7 gene (2^−ΔCt^). Relative gene expression in different tissues of blood-feeding female adults compared to those of sugar-feeding adults was calculated by using the Ct (2^−ΔΔCt^) method. The levels of DENV2 mRNA were determined using the RT-qPCR method as described previously [29]. Primers for real-time qPCR amplification were designed and are listed in Table S2 (Additional file 1: Table S2).

### Knockdown and overexpression of mosquito CTLs

Gene knockdown was performed via siRNA transfection. In brief, C6/36 cells were seeded into 12-well plates with serum-free media. Then, the cells were transfected with small interfering RNAs (siRNAs) using Lipofectamine 2000 Transfection Reagent (Invitrogen) according to the manufacturer’s protocol. At 48 h post-transfection, the cell samples were collected for qPCR or Western blotting analysis. Primers for siRNA were designed and are listed in Table S3 (Additional file 1: Table S3).

For overexpression of CTLs, the full-length cDNA of the *Ae. albopictus* CTL-encoding gene carrying a FLAG tag was amplified by PCR and then cloned and inserted into the vector pIB-V5 (Invitrogen). The recombinant pIB-CTL plasmid was confirmed by sequencing. Cells were seeded into 12-well plates overnight and transfected with pIB-CTL using TransIT DNA Transfection Reagent (Mirus). Cells transfected with the pIB-eGFP vector were used as controls. CTL protein expression was detected with a mouse anti-FLAG monoclonal antibody (GeneTex). Horseradish peroxidase-conjugated polyclonal rabbit anti-mouse IgG was used as a secondary antibody for Western blotting.

For the function assays of CTLs during DENV infection, C6/36 cells were transfected with siRNAs or overexpression plasmids for 12 h and then were infected with DENV2 as described above. The transfected-infected cells were cultured in the fresh growth medium for another 36 h. The cells were collected for subsequent analysis. Three wells were plated for each treatment, and the experiments were repeated in triplicate.

### Western blotting analysis

The samples were lysed in 150 μl RIPA lysis buffer supplemented with protease inhibitors. After centrifugation at 12,000 × g for 10 min, the supernatants were collected, and the protein concentrations were detected by using a BCA protein assay kit. Total protein samples were separated on 12% SDS-PAGE polyacrylamide gels and transferred to a polyvinyl difluoride (PVDF) membrane. After blocking with 5% milk in Tris-buffered solution containing Tween-20 (TBS-T), the PVDF membrane was labeled with primary antibodies that were separately used for detecting the DENV2 NS1 (1:1000) and FLAG tag (1:1000) overnight at 4 °C. Subsequently, HRP-conjugated goat anti-rabbit IgG (1:5000) secondary antibodies were added, and the membranes were incubated for 1 h at room temperature. The signals were measured with Supersignal West Pico Chemiluminescent Reagent (Pierce, USA) for 5 min. The densitometric analysis of the NS1 protein levels was performed using ImageJ software. The levels of the NS1 protein were separately normalized to that of β-actin. Independent experiments were carried out three times.

### Statistical analyses

Statistical analyses were performed by using one-way ANOVA with multiple comparison t-tests [30]. Gene expression values are presented as the mean ± SDs of at least three independent experiments. All statistical analyses were performed using GraphPad Prism 8 software (GraphPad Software Inc., CA): *p < 0.05, **p < 0.01, ***p < 0.005 and ****p < 0.001.

## Results

### Identification and bioinformatic analysis of CTL proteins in *Ae. albopictus*

**Fig. 1:**
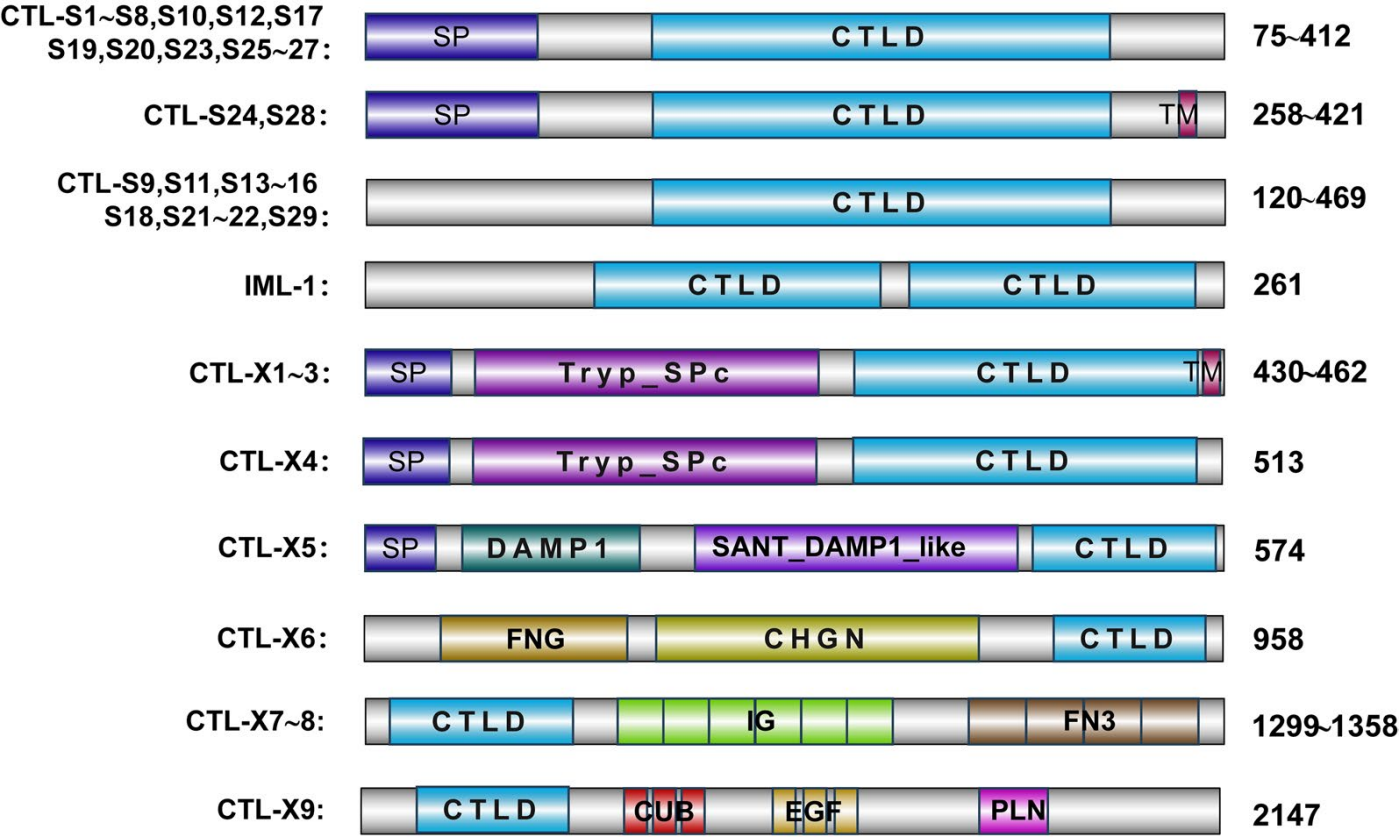
Domain architectures of 39 C-type lectins in *Aedes albopictus*. *SP* putative signal peptide, *CTLD* C-type lectin domain, *TM* transmembrane region, *Tryp_SPc* trypsin-like serine protease, *DAMP* damage-associated molecular pattern, *FN3* fibronectin type 3, *IG* immunoglobulin, *FNG* fringe, *CHGN* chondroitin N-acetylgalactosaminyltransferase, *EGF* epidermal growth factor-like, *CUB* extracellular domain, present in proteins mostly known to be involved in development, *PLN* translocon at the inner envelope of chloroplast subunit 62. Protein sizes or size ranges are shown to the right of each bar. The motif and protein sizes are not drawn to scale

We identified 39 potential CTL-encoding gene sequences in the *Ae. albopictus* (Foshan, FPA) genome sequence and annotated them in Table S1. The CTLs in *Ae. albopictus* were divided into three subfamilies according to the number of CTLDs and their structural characteristics. Twenty-nine CTLs containing a single CTLD were grouped into the CTL-S subfamily (CTL-S1 ~ S29). Only one CTL containing two CTLDs and no other conserved motif was assigned to the immulectin (IML) family, named IML-1. The remaining nine CTLs with both CTLD and other conserved domains were assigned to the CTL-X subfamily (CTL-X1 ~ 9) (Fig. 1).

The amino acid (aa) sequence lengths of *Ae. albopictus* CTLs vary from 75 to 2147 aa. Seventeen CTL-S proteins (CTL-S1 ~ 8, S10, S12, S17, S19 ~ 20, S23 and S25 ~ 27) and two CTL-X proteins (CTL-X4 and X5) contain an N-terminal signal peptide (SP) and are likely secreted into the plasma. Two CTL-S proteins (CTL-S24 and S28) and three CTL-X proteins (CTL-X1 ~ 3) contain both an N-terminal SP and a C-terminal transmembrane region (TM), suggesting that they may localize to the cell membrane. The remaining 15 CTL proteins (CTL-S9, S11, S13 ~ 16, S18, S21 ~ 22, S29, IML-1, X-6 ~ 9) without the N-terminal SP or C-terminal TM may be retained in the cytoplasm.

The CTLDs of 11 CTL proteins (CTL-S1, S7, S10, S12 ~ 14, S17, S19, S23 and X3 ~ 4) contain the Glu-Pro-Ser/Asn (EPS/N) tripeptide motif and belong to the mannose-binding type. The Gln-Pro-Asp (QPD) motif, which recognizes and binds to galactose ligands, was found in the CTLDs of nine CTL proteins (CTL-S8, S15, S20 ~ 22, S24, X1 ~ 2 and X5). We detected both EPS and QPD motifs in the CTLD of IML-1, indicating that it can bind with both mannose and galactose ligands (Table S1). Neither the EPS/N nor the QPD motif was found in the other 19 CTL proteins, and their characteristics need further investigation.

### Phylogenetic and structural features of CTLs

Considering the remarkable differences in structure among the CTL-S, IML and CTL-X subfamilies, the phylogenetic relationships of CTLs in *Ae. albopictus* and other mosquito species were separately analyzed. In the CTL-S subfamily, we constructed a phylogenetic tree of 29 CTL-S genes from *Ae. albopictus* and 43 CTL-S genes from *Ae. aegypti*. CTL-S was divided into three clades according to the bootstrap values (Fig. S1a). Clade A contained six CTL-S genes (CTL-S3, S8, S15 ~ 16, S18 and S23) in *Ae. albopictus* and 13 CTL-S genes in *Ae. aegypti*. Eight CTL-S genes (CTL-S1, S4 ~ 5, S7, S12 ~ 14 and S17) in *Ae. albopictus* and 14 CTL-S genes in *Ae. aegypti* are classified into the clade B. The remaining 15 CTL-S genes (S2 S6, S9 ~ 11, S19 ~ 22, S24 ~ 29) in *Ae. albopictus* and 16 CTL-S genes in *Ae. aegypti* are located in clade C. IML-1 in *Ae. albopictus* exhibited high bootstrap values with four CTLs in* Cx. quinquefasciatus* (Fig.S1b). No homologous IML gene was detected in *Ae. aegypti*. With the exception of CTL-X6 gene, which is located in an independent clade without a homologous gene, the other eight CTL-X genes in *Ae. albopictus* all have homologous CTL genes from *Ae. aegypti* (Fig. S1c).

**Fig. 2:**
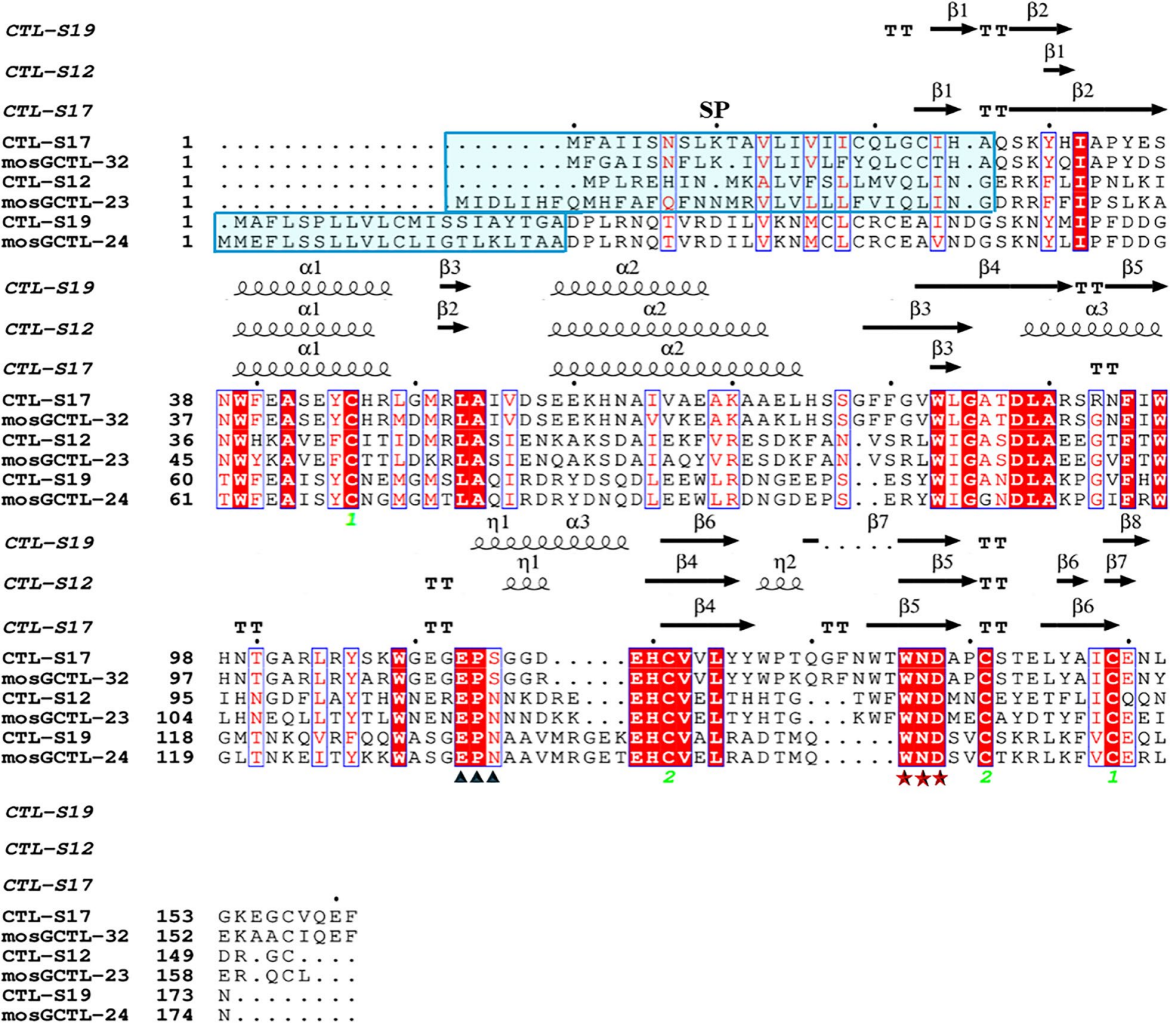
Amino acid sequence alignment of CTL in *Aedes* mosquitoes. Alignments of CTL in *Aedes albopictus* (CTL-S12, S17 and S19) and *Ae. aegypti* (mosGCTL-23, mosGCTL-24 and mosGCTL-32) were performed by using ESPript 3.0 software. Red highlights indicate all identical residues; blue bold texts indicate the majority of conserved residues. The carbohydrate recognition sites are marked with black triangles, and the Ca2^+^-interacting residues are marked with red stars below the sequences. The putative signal peptide (SP) sequences are highlighted in cyan. The secondary structure elements (α-helix, β-sheet and turn) of CTLs are shown on top of the alignment. The green numbers below the conserved cysteine residues (Cys1 and Cys2) indicate that two disulfide bonds formed

**Fig. 3:**
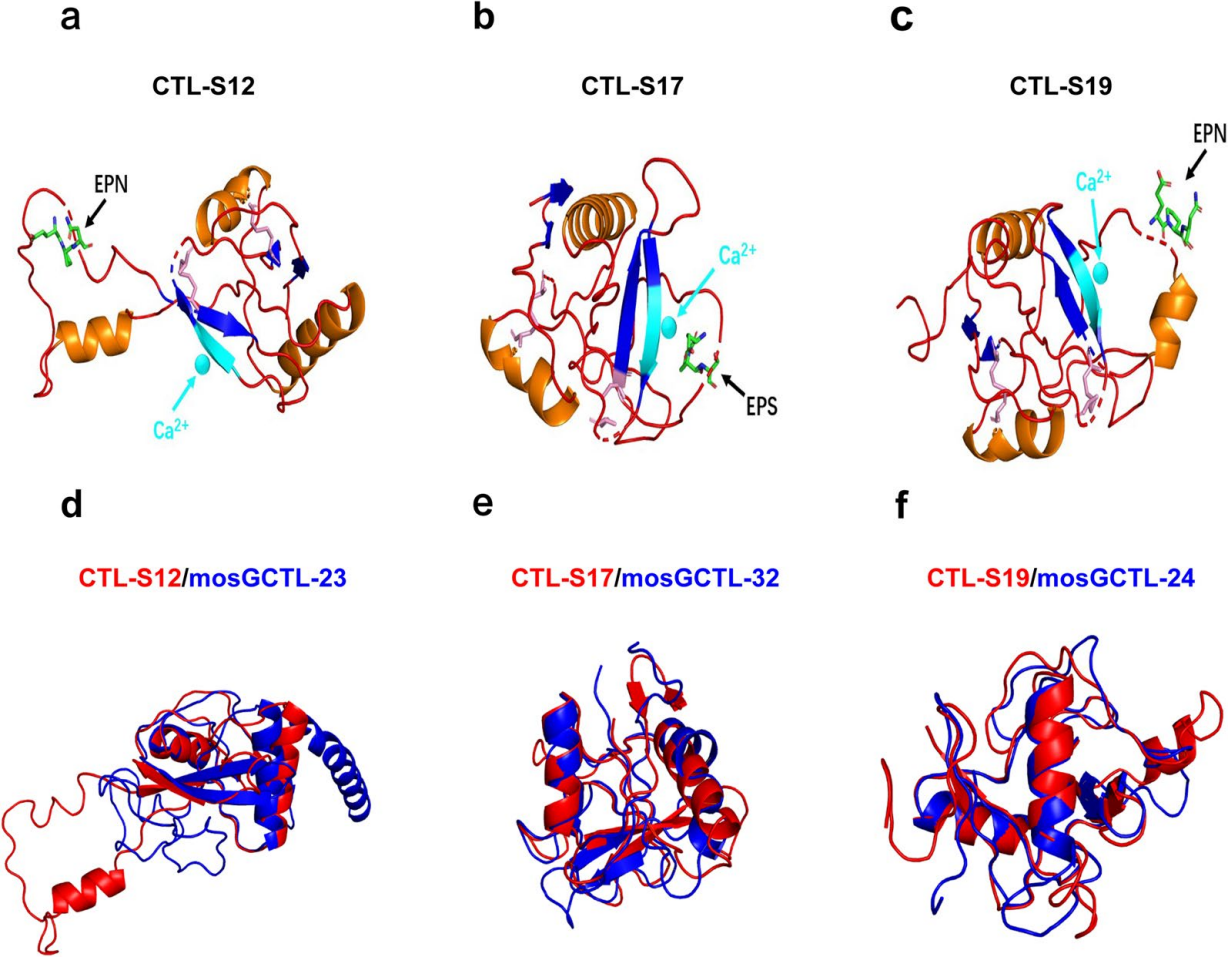
Tertiary structures of the CTLs in *Aedes* mosquitoes. Structural models of CTL-S12 **a**, CTL-S17 **b** and CTL-S19 **c** were predicted from the I-TASSER server and are shown as cartoons. The calcium ions are shown as cyan spheres and are indicated by cyan arrows. The EPN/S motif is shown as green sticks and indicated by black arrows. The cysteine residues for the formation of disulfide bonds are represented as pink sticks. The superimpositions of CTL-S12 and mosGCTL-23 **d**, CTL-S17 and mosGCTL-32 **e** and CTL-S19 and mosGCTL-24 **f** are colored red and blue, respectively

Previous studies confirmed that knockdown of three CTLs (mosGCTL-23, mosGCTL-32 and mosGCTL-24) individually reduced DENV replication in *Ae. aegypti* [28]. We found that three CTL-S genes (S12, S17 and S19) from *Ae. albopictus* in the phylogenetic trees were closely related to AEL011455 (mosGCTL-23), AAEL012353 (mosGCTL-32) and AAEL014382 (mosGCTL-24) from *Ae. aegypti* (Fig. S1a). The above six CTLs contain the signal peptides in the N-terminus of amino acid sequences, indicating that they are secreted proteins (Fig. 2). These CTLs share conserved 2D structural characteristics, including α-helixes, β-sheets and two pairs of disulfide bonds in the CTLDs. We also found the EPN/EPS and the WND motifs in the CTLDs that respond to mannose-binding and Ca^2+^-bound sites, respectively. The co-occurrence of these conserved residues might be strongly implicated in calcium-dependent carbohydrate binding. We predicted the 3D structures of the six CTLs and the potential Ca^2+^ and sugar binding sites (Fig. 3). These results showed that the functional domain architectures of CTLs are conserved in both *Ae. albopictus* and *Ae. aegypti*.

### Expression characteristic of CTL genes in *Ae. albopictus*

**Fig. 4:**
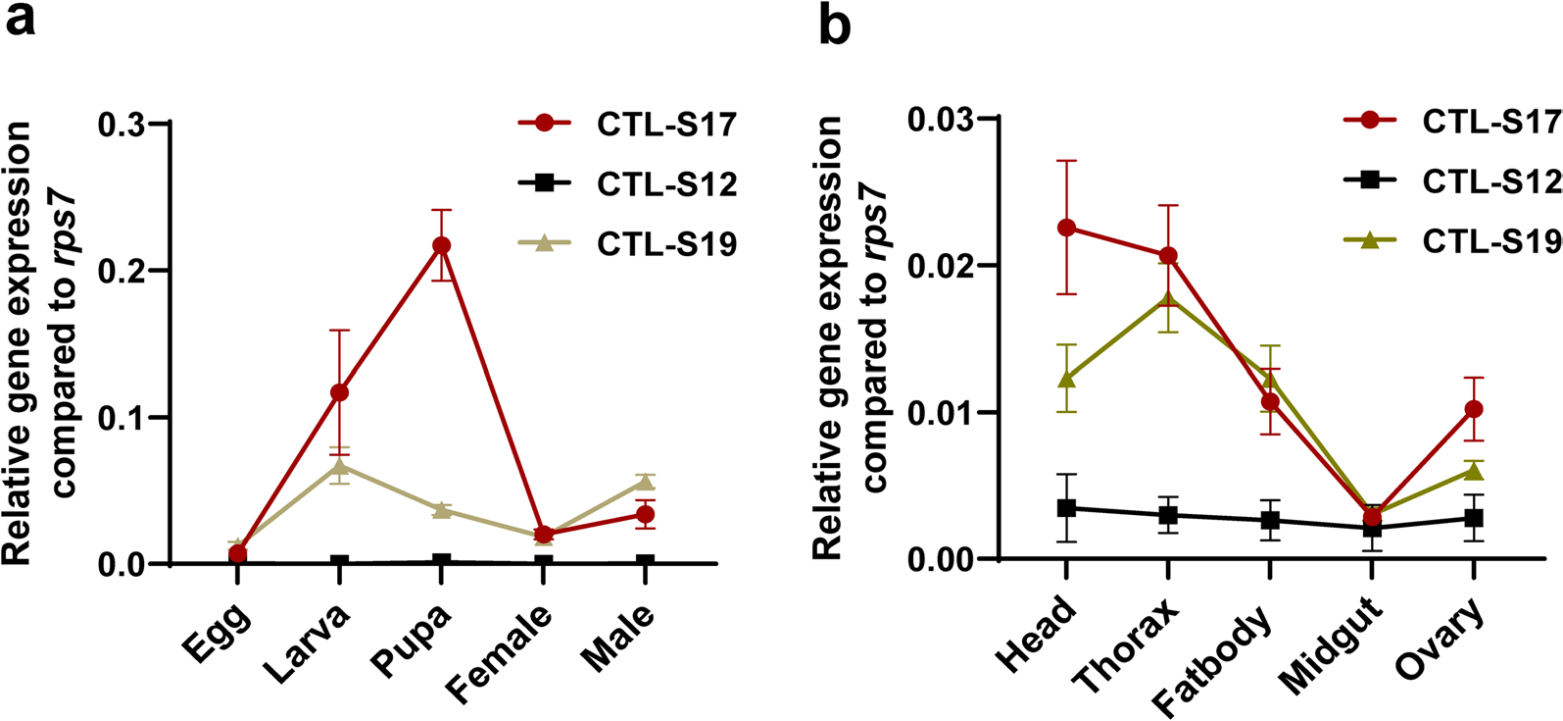
Expression patterns of the *Aedes albopictus* CTL genes at different developmental stages **a** and in adult female tissues **b**. RNA was prepared from five specimens of eggs, larvae (3 ~ 4 instars), pupae, sugar-feeding females and males (all adult mosquitoes were collected on the same day; 3 days post eclosion). The different tissues of female adults including the head, thorax, fatbody, midgut and ovary were separately dissected on ice. Mosquito samples with 20 individuals in each treatment were placed into 1.5-ml sterile Eppendorf tubed containing 250 µl PBS buffer and homogenized in ice for 1 min. The samples were subjected to RT-qPCR to assess the relative expression of CTLs. RNA levels between samples were normalized to those of the rps7 gene using the 2^-ΔCt^ method. The data represent three biological replicates in each treatment

We investigated the temporal expression patterns of the CTL-S12, S17 and S19 genes in different developmental stages of *Ae. albopictus* mosquitoes via relative expression analyses. The expression level of the CTL-S12 gene remained relatively stable from the egg to adult stages and was much lower than that of CTL-S17 and S19 (Fig. 4a). We detected a much higher level of expression for both the CTL-S17 and S19 genes in the postembryonic development stages than in the egg stage. The highest expression level of the CTL-S17 gene was detected in the pupa stage, after which it decreased in the adult stage. The expression level of the CTL-S19 gene in the larval stage was much higher than that in the other three stages: the egg, pupa and adult stages. None of the above three CTL genes exhibited significant differences in the expression between the male and female adults (Fig. 4a).

**Fig. 5:**
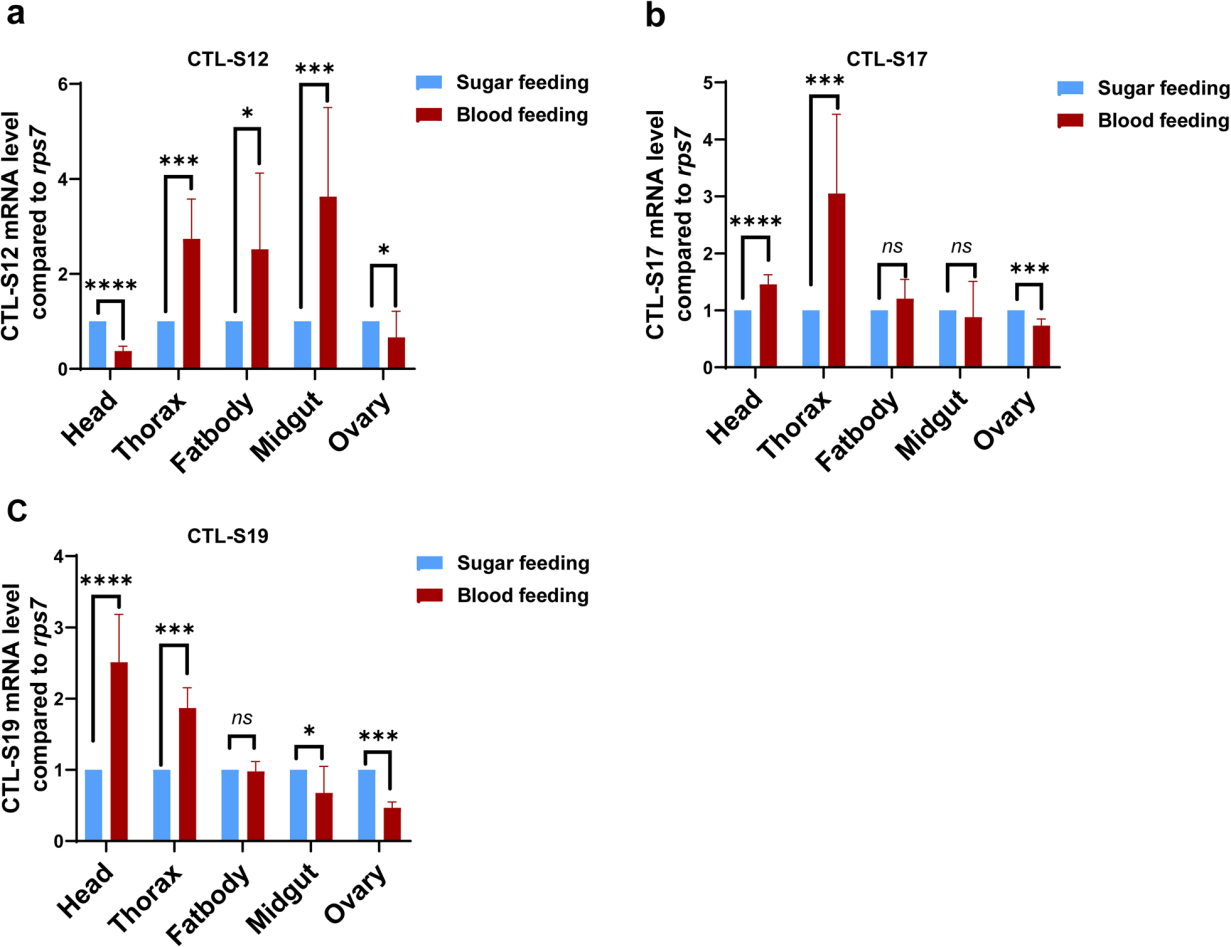
Expression patterns of CTL genes in female adults after sugar feeding and blood feeding. Relative gene expression levels of CTL-S12 **a**, CTL-S17 **b** and CTL-S19 **c** in the head, thorax, fat body, midgut and ovary in the sugar feeding and 24-h post blood feeding groups. RNA levels between samples were normalized to those of the rps7 gene using the 2^−ΔΔCt^ method. The data represent three biological replicates with 20 individuals in each and are shown as the mean ± SEM. *p < 0.05, **p < 0.01, ***p < 0.005, ****p < 0.001

The tissue expression patterns of three CTL genes (CTL-S12, S17 and S19) in female adults were also examined. We observed that all three CTL genes in the midgut exhibited much lower expression levels than those in the other tissues, such as the head, thorax, fatbody and ovary (Fig. 4b). Both the CTL-S17 and S19 genes exhibited high-level expression in the head and thorax. The expression level of the CTL-S12 gene did not significantly differ among the different tissues of female adults. To explore whether the expression patterns of CTLs are affected by the diet switch from sugar to blood, we collected female adults after sugar and blood feeding individually and investigated the expression patterns of CTLs in the female mosquito tissues. The expression level of the CTL-S12 gene increased in the thorax, fatbody and midgut but decreased in the head and ovary (Fig. 5a). Blood feeding induced the expression of both CTL-S17 and S19 genes to be upregulated in head and thorax but downregulated in the ovary (Fig. 5b, c).

### Functional analysis of CTLs in mosquito cells after DENV infection

To investigate the functional role of CTLs in response to DENV2 infection, we first assayed the expression patterns of the CTL-S12, S17 and S19 genes in DENV2-infected C6/36 cells at 24 h.p.i. and 48 h.p.i. DENV2 infection significantly upregulated the expression of the CTL-S12 gene at 24 h.p.i. compared to that in the uninfected cells (Additional file 1: Fig. S2a). Similar expression upregulation of the CTL-S17 and S19 genes were also observed in DENV2-infected C6/36 cells at 24 h.p.i. or/and 48 h.p.i (Additional file 1: Fig. S2b and c).

**Fig. 6:**
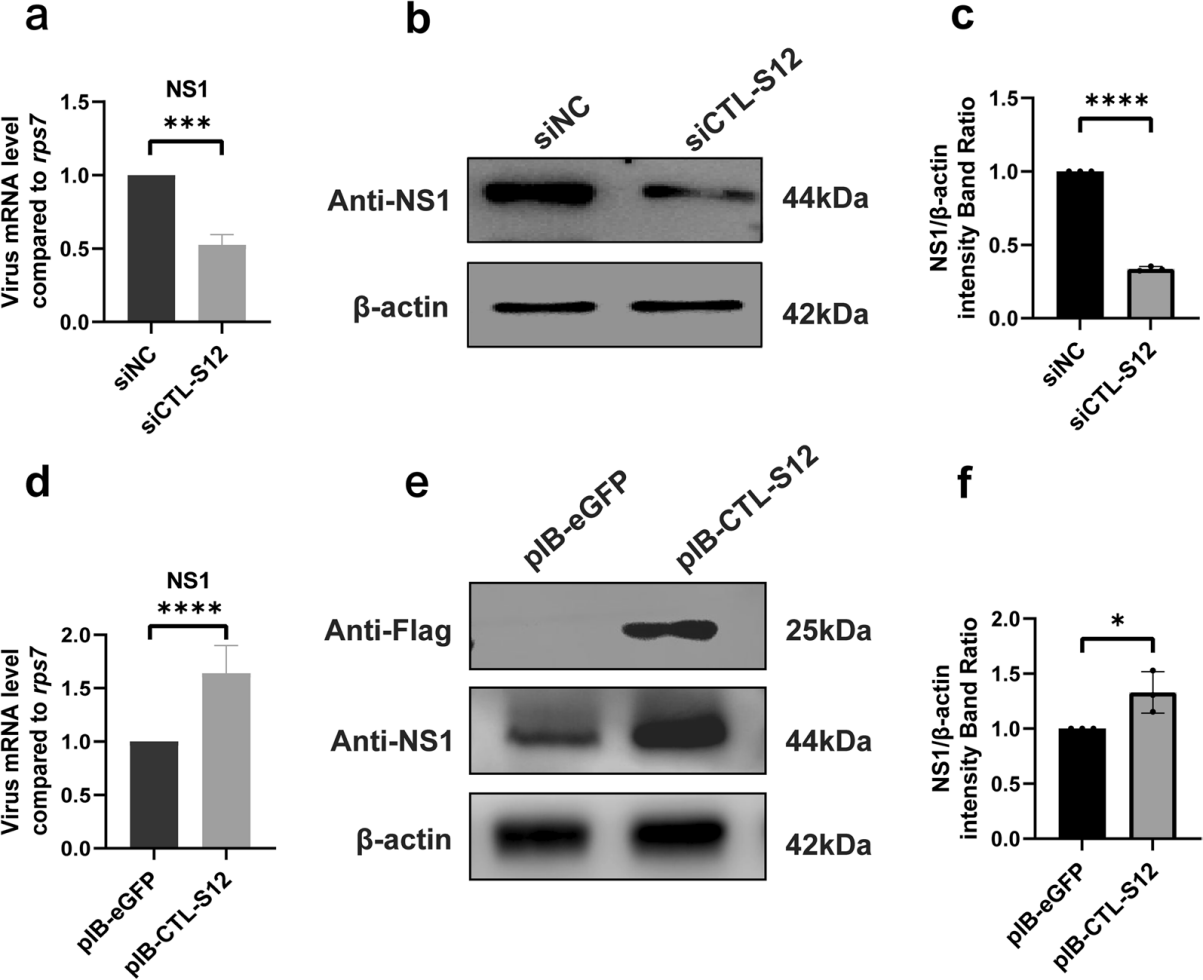
Functional analysis of CTL-S12 in C6/36 cells after DENV2 infection. After silencing of the CTL-S12 gene by siRNA transfection, the titer of DENV2 was analyzed for both viral RNA replication by RT-qPCR **a** and protein production by Western blot **b**. After overexpression of the CTL-S12 gene by plasmid transfection, the titer of DENV2 was analyzed for both viral RNA replication by RT-qPCR **d** and protein production by Western blot **e**. eGFP siRNA and pIB-eGFP vector transfection served as the siRNA negative controls (siNC) and overexpression plasmid controls, respectively. β-Actin was used to normalize the levels of protein loading by using densitometric analyses. **c** and **f** The ratios of NS1 to actin were determined by using band density analysis. The data are presented as the means ± SEMs of three independent experiments; *p < 0.05, ***p < 0.005, ****p < 0.001

**Fig. 7:**
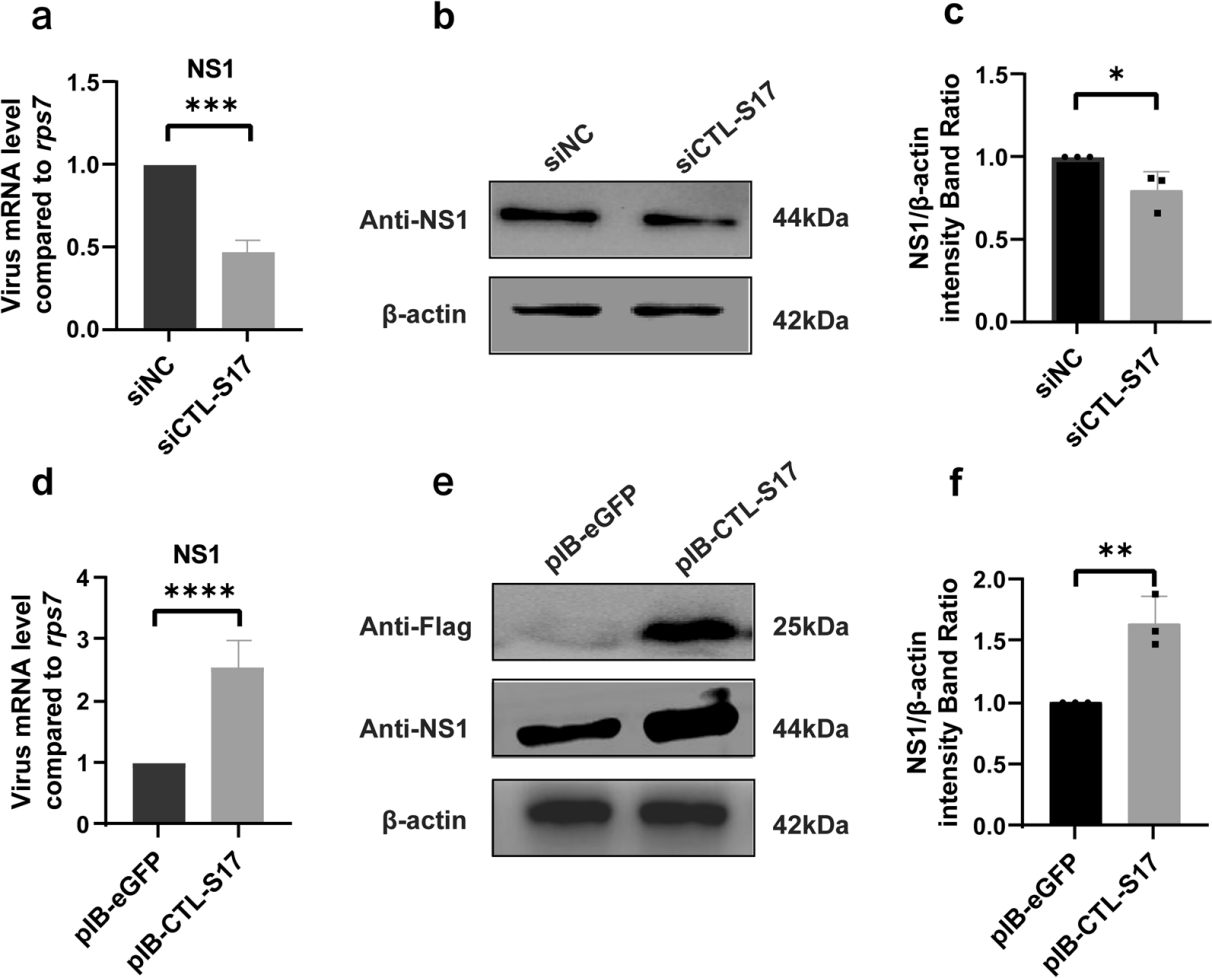
Functional analysis of CTL-S17 in C6/36 cells after DENV2 infection. After silencing of the CTL-S17 gene by siRNA transfection, the titer of DENV2 was analyzed for both viral RNA replication by RT-qPCR **a** and protein production by Western blot **b**. After overexpression of the CTL-S17 gene by plasmid transfection, the titer of DENV2 was analyzed for both viral RNA replication by RT-qPCR **d** and protein production by Western blot **e**. eGFP siRNA and pIB-eGFP vector transfection served as the siRNA negative controls (siNC) and overexpression plasmid controls, respectively. β-Actin was used to normalize the levels of protein loading by using densitometric analyses. **c** and **f** The ratios of NS1 to actin were determined by using band density analysis. The data are presented as the means ± SEMs of three independent experiments; *p < 0.05, **p < 0.01, ***p < 0.005, ****p < 0.001

**Fig. 8:**
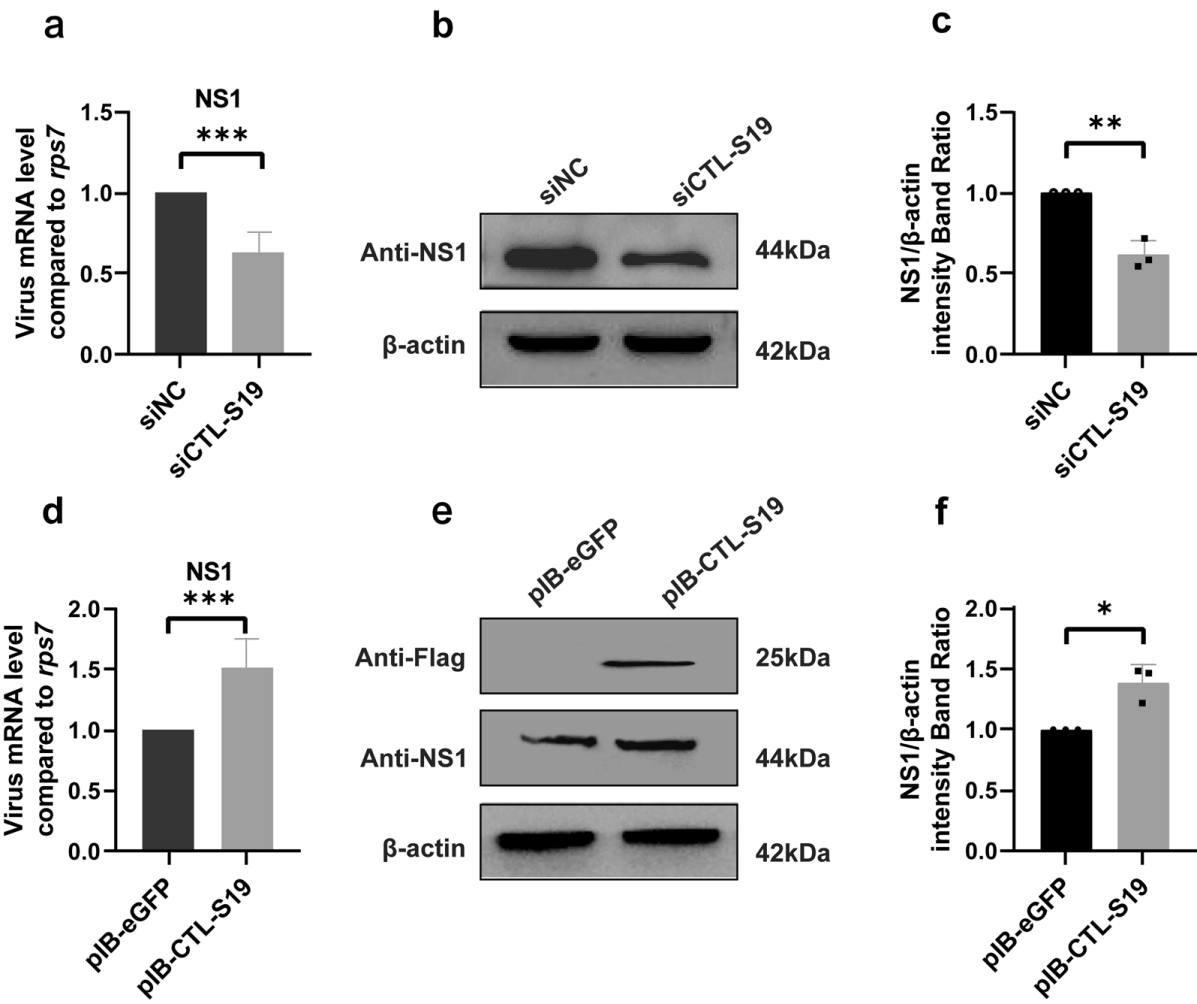
Functional analysis of CTL-S19 in C6/36 cells after DENV2 infection. After silencing of the CTL-S19 gene by siRNA transfection, the titer of DENV2 was analyzed for both viral RNA replication by RT-qPCR **a** and protein production by Western blot **b**. After overexpression of the CTL-S19 gene by plasmid transfection, the titer of DENV2 was analyzed for both viral RNA replication by RT-qPCR **d** and protein production by Western blot **e**. eGFP siRNA and pIB-eGFP vector transfection served as the siRNA negative controls (siNC) and overexpression plasmid controls. β-Actin was used to normalize the levels of protein loading by using densitometric analyses. **c** and **f** The ratios of NS1 to actin were determined by using band density analysis. The data are presented as the means ± SEMs of three independent experiments; *p < 0.05. **p < 0.01, ***p < 0.005

Subsequently, knockdown of the CTL-S12, S17 and S19 genes was individually carried out by using a siRNA strategy. After the transfection of CTL-S12 siRNA oligos into C6/36 cells, we observed a significant reduction in the mRNA level of CTL-S12 (Additional file 1: Fig. S3). The DENV2 infection titers were then analyzed in the CTL-S12 knockdown cells (Fig. 6a–c). We observed significant decreases in both viral mRNA replication and the NS1 protein expression in the DENV2-infected cells after CTL-12 siRNA treatment compared to the siRNA negative control (siNC). Similar reductions in DENV2 RNA replication and protein expression were found after both CTL-S17 siRNA (Fig. 7a–c) and S19 siRNA treatment (Fig. 8a–c). These results indicated that the knockdown of the above three CTLs by siRNA transfection reduced DENV infection in the mosquito cells.

Finally, the recombinant plasmid pIB-CTL harboring the *Ae. albopictus* CTL-S12, S17 and S19 genes was transfected into C6/36 cells, respectively. A FLAG tag was fused to the COOH terminus of each CTL gene for the Western blot analysis. CTL overexpression was confirmed in C6/36 cells by using Western blotting with an anti-FLAG monoclonal antibody. Compared with that of cells transfected with pIB-eGFP, viral mRNA replication in CTL-S12-overexpressing cells significantly increased following DENV2 infection (Fig. 6d). Furthermore, the NS1 protein level in DENV2-infected cells was also increased after CTL-S12 overexpression (Fig. 6e, f). Similar increases in DENV2 RNA replication and protein expression were found in both CTL-S17-overexpressing cells (Fig. 7d–f) and S19-overexpressing cells (Fig. 8d–f). These results suggested that genetic activation of CTLs promotes DENV infection in *Ae. albopictus* mosquito cells.

## Discussion

Insects live in complex environments full of diverse microorganisms, including bacteria, fungi, viruses and parasites [14]. CTLs constitute a group of evolutionarily conserved proteins with sugar-binding activity and act as pathogen-specific recognition molecules in both invertebrates and vertebrates [31, 32]. Genome-wide analyses in insects have shown that remarkable CTL gene expansion can increase the ability of hosts to recognize diverse pathogenic microorganisms [20]. In this study, we identified 39 CTL-encoding genes in the *Ae. albopictus* genome, which is fewer than the number of CTLs in *Ae. aegypti* (52 CTLs) and *C. quinquefasciatus* (64 CTLs) [23]. More than 74% of CTLs belong to the CTL-S subfamily, which contains only a single CTLD. In addition to IML-1 and CTL-X6, orthologs of CTLs were found in both *Ae. albopictus* and *Ae. aegypti*, indicating the phylogenetic conservation of CTL genes in *Aedes* mosquito species.

Most insect CTL-Ss only contain one type of carbohydrate-binding motif (EPN/S or QPD) in the CTLD [21, 22, 33]. The sugar-binding specificity of CTLs is determined by several conserved residues in CTLD, which are coordinated with Ca^2+^ to form the basis of a primary sugar-binding site [17]. IML-1, which is only present in *Ae. albopictus* but not in *Ae. aegypti*, contains both QPD and EPN motifs in the two CTLDs, suggesting that it can bind both mannose and galactose ligands. A variety of IMLs have also been identified in *Bombyx mori* [22], *Ostrinia furnacalis* [34] and *Manduca sexta* [33]*.* In addition to the CTLDs, four CTL-Xs (CTL-X1–X4) contain a trypsin-like serine protease (Tryp_SPc) domain. Trypsin-like serine peptidases are mostly located in mosquito midguts [35, 36] and play pivotal roles in oogenesis, immunity, metamorphosis and the modulation of embryonic development and nutrition [37]. Furthermore, the secretion of trypsin-like serine peptidases into the lumen of the midgut is involved in defense against pathogens [28, 29]. Thus, it will be meaningful to investigate the function of CTL-X in *Ae. albopictus* in the future.

Temporospatial expression of CTL genes reflects the potential function of their protein products in insect development [33]. For example, a CTL from the red flour beetle TcCTL14 was highly expressed in the late pupae and central nervous system of *Tribolium castaneum* [38]. Knockdown of TcCTL14 suppressed metamorphosis, reduced fecundity and delayed embryogenesis [38]. We found that all three CTL-S genes (CTL-S12, S17 and S19) exhibited relatively low expression in the egg stages of *Ae. albopictus*, which is consistent with the CTL-S gene expression pattern in the embryonic stage of *Ae. aegypti* [23]. The expression level of the CTL-S17 gene is upregulated in the larval stage and continues through pupal development, with reduced expression in the adult stage. The expression pattern of the homologous gene AAEL012353 in *Ae. aegypti* was similar to that of CTL-S17 [23]. CTL-S19 in *Ae. albopictus* and the homologous gene AAEL014382 in *Ae. aegypti* were highly expressed in the larval stage. In the cotton bollworm, depletion of C-type lectin reduces larval body size and delays pupation time, which suggests that CTLs are involved in maintaining normal larval growth and development [39].

Female mosquitoes need to feed on vertebrate blood for egg maturation. In our study, blood meals increased the expression of all three CTL-S genes in the thorax but decreased it in the ovary. Downregulated expression of CTL-Ss was also observed in the ovaries of *Ae. aegypti* after blood feeding [23]. In *Ae. aegypti*, mutants of CTL (mosGCTL-3) cause significantly shorter lifespans and fewer eggs because of defective germline development [40]. We also observed that all three CTL genes in the midgut exhibited relatively low expression levels before blood feeding. Blood meals significantly increased the expression level of the CTL-S12 gene but decreased CTL-S19 gene expression in the midgut.

The midgut not only acts as a predominant route for food digestion and nutrient absorption but also is also a pivotal entry site for DENV [10, 11]. As the primary vectors for the transmission of many arboviruses, mosquitoes may have developed a general mechanism to facilitate viral invasion [12, 26, 28]. Several C-type lectins were identified as ligands that interact with arboviruses during infection of both mammalian hosts and mosquito vectors [26, 27]. In mammalian cells, multiple C-type lectins, including DC-SIGN (a C-type lectin in dendritic cells), the mannose receptor and C-type lectin domain family 5 (CLEC5), recognize glycans on the surface of arboviruses to facilitate viral entry or contribute to inflammation-related pathogenesis [41–43]. In mosquito cells, a mosquito C-type lectin, mosGCTL-1, is a receptor that cooperates with a membrane-bound protein (mosPTP-1) to enhance WNV infection [27]. DENV has been shown to interact with a variety of mosCTLs to facilitate DENV-2 invasion [28]. We found that DENV2 infection significantly upregulated the expression of 3 CTL-S genes. Silencing of the CTL-S12, S17 and S19 genes individually resulted in a significant reduction in the mRNA and protein levels of DENV2. In contrast, the overexpression of CTLs enhances DENV infection in mosquito cells. The mosGCTLs in *Ae. aegypti* interact directly with the DENV-2 E protein efficiently to enhance viral infection [28]. Knockdown of the homolog 3 CTLs (mosGCTL-23, mosGCTL-32 and mosGCTL-24) individually reduced DENV replication [28]. We reasoned that CTL-S in *Ae. albopictus* may also recognize the E protein of DENV and play a role in enhancing DENV infection. Our studies have indicated that DENV may employ conserved CTLs in both *Ae. albopictus* and *Ae. aegypti* to promote virus infection.

## Conclusions

Here, we characterized the molecular features of CTL gene families in *Ae. albopictus* and the functional role of CTLs in response to DENV infection. Our study confirmed 39 genes encoding CTL proteins in the *Ae. albopictus* genome assembly. Sequence homology suggested the high conservation of CTLs from *Ae. albopictus* and *Ae. aegypti*, and the presence of a mannose-type EPN motif or galactose-type QPD motif in the CTL domains supports the ability of CTLs to recognize and bind carbohydrate ligands. The differential expression characteristics revealed the potentially important function of CTL genes in regulating developmental and physiological processes. In addition, these findings demonstrated that CTLs promote dengue virus infection, which provides a novel target for developing a novel strategy for blocking virus transmission in *Ae. albopictus* mosquitoes.

## Supplementary Information

Additional file 1: **Figure S1.** Phylogenetic analysis of CTLs in *Aedes albopictus* and other mosquito species by using the neighbor-joining method. **Figure S2. **Transcription levels of CTL genes were detected in C6/36 cells infected with DENV2 for 24 and 48 h by RT-qPCR. **Figure S3.** Knockdown of CTL genes in C6/36 cells by siRNA transfection. **Table S2.** Primers for genes expression analyses in RT-qPCR.** Table S3.** siRNA sequences for knockdown studies.

Additional file 2: **Table S1.** Features of the CTLs in *Aedes albopictus*.

## Data Availability

No datasets were generated or analyzed during the current study.

## Abbreviations

*Ae*. *albopictus*: *Aedes* *albopictus*
*Ae*. *aegypti*: *Aedes* *aegypti*
*An*. *gambiae*: *Anopheles* *gambiae*
*B*. *mori*: *Bombyx* *mori*
CTL: C-type lectin
CTLD: C-type lectin domain
DENV: Dengue virus
MOI: Multiplicity of infection
h.p.i: Hours post infection
RT-qPCR: Reverse transcription-quantitative PCR
rps7: Ribosomal S7
siRNAs: Interfering RNAs
PVDF: Polyvinyl difluoride
SP: Signal peptide
TM: Transmembrane region
