# Supplementary material for: Identification and functional analysis of C-type lectin from mosquito Aedes albopictus in response to dengue virus infection
Source: Parasit Vectors. 2024 Sep 4;17:375. doi: 10.1186/s13071-024-06453-9 (PMC11373435; doi:10.1186/s13071-024-06453-9)
Supplement: Supplementary file 1 — Additional file 1: Figure S1. Phylogenetic analysis of CTLs in Aedes albopictus and other mosquito species by using the neighbor-joining method. Figure S2. Transcription levels of CTL genes were detected in C6/36 cells infected with DENV2 for 24 and 48 h by RT-qPCR. Figure S3. Knockdown of CTL genes in C6/36 cells by siRNA transfection. Table S2. Primers for genes expression analyses in RT-qPCR. Table S3. siRNA sequences for knockdown studies. [file 13071_2024_6453_MOESM1_ESM.docx]

**Additional file**

**Supplemental Figure**

**Fig. S1** Phylogenetic analysis of CTLs in *Ae. albopictus* and other mosquito species by using the neighbor-joining method. Members belonging to *Ae. albopictus* CTL-S1~29 (**a**), IML-1 (**b**) and CTL-X1~9 (**c**) were separately aligned with their orthologs from 2 other mosquito species: *Ae. aegypti* (AAEL) and *C. quinquefasciatus* (CPIJ). The scale bar indicates 10% nucleotide sequence divergence.

**
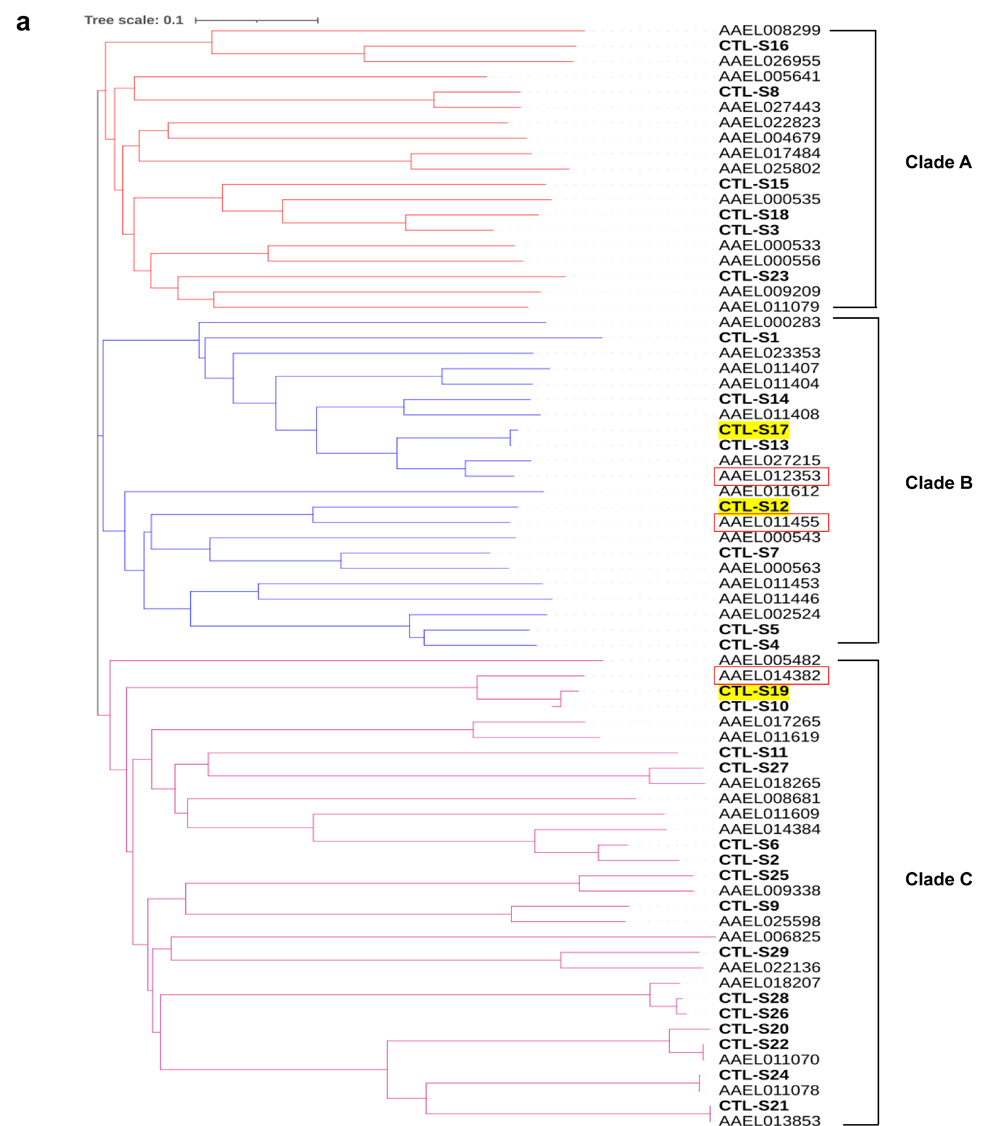
**

**
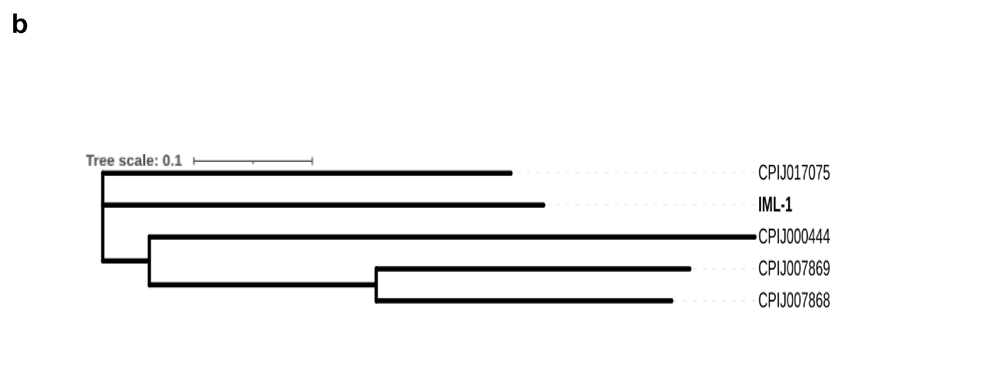
**

**
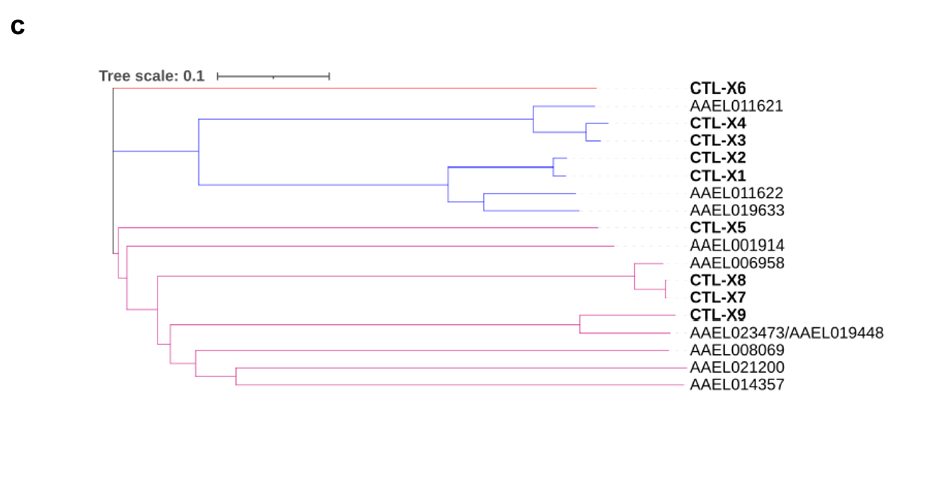
**

**Fig. S2 Transcription levels of CTL genes were detected in C6/36 cells infected with DENV2 for 24 and 48 h by RT‒qPCR.** CTL-S12 (**a**); CTL-S17 (**b**); CTL-S19 (**c**). C6/36 cells were used as the mock control, and RNA levels between samples were normalized to those of the rps7 gene using the 2^−ΔΔCt^ method. The data represent three independent replicates and are shown as the mean ± SEM. *p < 0.05, ***p < 0.005, ****p < 0.001.

**
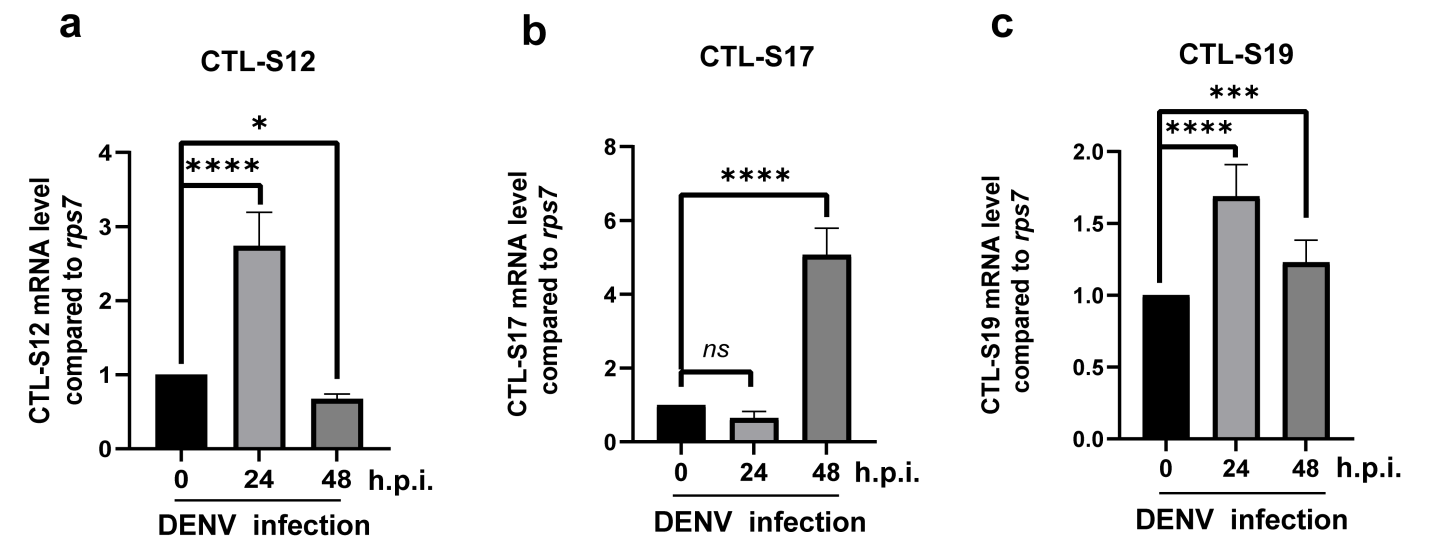
**

**Fig. S3 Knockdown of CTL genes in C6/36 cells by siRNA transfection**. CTL-S12 (**a**); CTL-S17 (**b**); CTL-S19 (**c**).C6/36 cells transfected with siRNA for knockdown of GFP were used as the siControl, and RNA levels between samples were normalized to those of the rps7 gene using the 2^−ΔΔCt^ method. The data represent three independent replicates and are shown as the mean ± SEM. *****p* < 0.001.


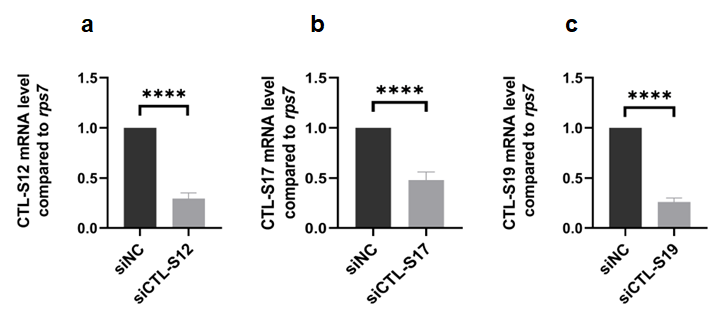


**Supplemental Tables**

**Table S2. Primers for genes expression analyses in RT-qPCR**

| **Target gene** | **Primers** |
| --- | --- |
| CTL-S12 | F: 5’- ACATGAAGGCTCTCGTGTTCT-3’  R: 5’ -ACCGCCTTGTGCCAGTTTAT-3’ |
| CTL-S17 | F: 5’- CGATCTGGCTCGGTCAAGAA -3’  R: 5’-GCGTAGGCCAGTAGTACAGC- 3’ |
| CTL-S19 | F: 5’-GCCACTCTTAGTACTTTGCATGAT-3’  R: 5’- GATGTCTCGCACAGTTTGATTC-3’ |
| DENV2-NS1 | F: 5’ -AACGCTGAAGGACAAGCCAAAC-3’  R: 5’-ACGGCAACATTCCTCCACGATA-3’ |
| RPS7 | F: 5’- GAAGTTGTCGGAAAGCGTATGC-3’  R: 5’ -TTCAATGGTGGTCTGCTGGTTC-3’ |

Note: F: Forward primer; R: Reverse primer.

**Table S3. siRNA sequences for knock down studies.**

| **Target gene** | **siRNA sequences (**5’-3’) |
| --- | --- |
| CTL-S12 | AACAAGGACCGUGAAGAAC(dT)(dT) |
| CTL-S17 | UGAAGUUUCUUGACCGAGC(dT)(dT) |
| CTL-S19 | UCGAAGCGAUUUCAUACUG(dT)(dT) |
| Negative control | CAGUAUGAAAUCGCUUCGA(dT)(dT) |
